# C_60_ Fullerenes Diminish Muscle Fatigue in Rats Comparable to *N*-acetylcysteine or β-Alanine

**DOI:** 10.3389/fphys.2018.00517

**Published:** 2018-05-15

**Authors:** Inna V. Vereshchaka, Nataliya V. Bulgakova, Andriy V. Maznychenko, Olga O. Gonchar, Yuriy I. Prylutskyy, Uwe Ritter, Waldemar Moska, Tomasz Tomiak, Dmytro M. Nozdrenko, Iryna V. Mishchenko, Alexander I. Kostyukov

**Affiliations:** ^1^The Unit of the Theory of Physical Education, The Chair of Physical Education, Gdansk University of Physical Education and Sport Gdańsk, Poland; ^2^Department of Movement Physiology, Bogomoletz Institute of Physiology, National Academy of Sciences, Kyiv, Ukraine; ^3^Department of Hypoxic States Investigation, Bogomoletz Institute of Physiology, National Academy of Sciences, Kyiv, Ukraine; ^4^ESC “Institute of Biology and Medicine", Taras Shevchenko National University of Kyiv, Kyiv, Ukraine; ^5^Institute of Chemistry and Biotechnology, Technical University of Ilmenau, Ilmenau, Germany; ^6^Lesya Ukrainka East European National University, Lutsk, Ukraine

**Keywords:** C_60_ fullerene, skeletal muscles fatigue, electrical stimulation, oxidative stress markers, antioxidant system

## Abstract

The aim of this study is to detect the effects of C_60_ fullerenes, which possess pronounced antioxidant properties, in comparison with the actions of the known exogenous antioxidants *N*-acetylcysteine (NAC) and β-Alanine in terms of exercise tolerance and contractile property changes of the *m. triceps surae* (*TS*) during development of the muscle fatigue in rats. The electrical stimulation of the *TS* muscle during four 30 min series in control rats led to total reduction of the muscle contraction force. Furthermore, the effects of prior intraperitoneal (i.p.) or oral C_60_FAS application and preliminary i.p. injection of NAC or β-Alanine on muscle contraction force under fatigue development conditions is studied. In contrast to control rats, animals with C_60_FAS, NAC, or β-Alanine administration could maintain a constant level of muscle effort over five stimulation series. The accumulation of secondary products and changes in antioxidant levels in the muscle tissues were also determined after the fatigue tests. The increased levels of lactic acid, thiobarbituric acid reactive substances and H_2_O_2_ after stimulation were statistically significant with respect to intact muscles. In the working muscle, there was a significant (*p* < 0.05) increase in the activity of endogenous antioxidants: reduced glutathione, catalase, glutathione peroxidase, and superoxide dismutase. Treated animal groups showed a decrease in endogenous antioxidant activity relative to the fatigue-induced animals (*P* < 0.05). Oral C_60_FAS administration clearly demonstrated an action on skeletal muscle fatigue development similar to the effects of i.p. injections of the exogenous antioxidants NAC or β-Alanine. This creates opportunities to oral use of C_60_FAS as a potential therapeutic agent. Due to the membranotropic activity of C_60_ fullerenes, non-toxic C_60_FAS has a more pronounced effect on the prooxidant-antioxidant homeostasis of muscle tissues in rats.

## Introduction

Fullerenes are a new kind of organic compounds, consisting of carbon atoms with very attractive photo-, electrochemical and physical properties that can be used in many biological fields, which make fullerenes potential therapeutic agents for a wide range of applications in nanomedicine (Anilkumar et al., 2011; Dellinger et al., 2013; Castro et al., 2017). With respect to its electron donor and acceptor capability fullerenes can be effective antioxidants and radical scavengers (Bosi et al., 2003; Bakry et al., 2007). It was shown that under the influence of light, fullerene acts as a prooxidant (Kamat et al., 2000), generating singlet oxygen (Bosi et al., 2003), which can be used in photodynamic therapy of cancer and other diseases. Some fullerene derivatives exhibit inhibitory activity against human immunodeficiency virus reverse transcriptase and hepatitis C virus RNA polymerase (Mashino et al., 2005; Nakamura and Mashino, 2012), and can stabilize immune effector cells to prevent or inhibit the release of proinflammatory mediators, making them potential candidates for a variety of diseases, such as asthma, arthritis, and multiple sclerosis (Dellinger et al., 2013). Thus, fullerenes and their derivatives are excellent candidates for multiple functionalization.

It is known, that muscle fatigue is accompanied by ionic changes in action potentials (Allen et al., 2008) along with various metabolic disturbances in skeletal muscles, when reactive oxygen species (ROS) are formed (Halliwell and Gutteridge, 1989), excess lactic acid (LA) (Sahlin et al., 1987), and lipid peroxidation (Venditti and Di Meo, 1997). With excessive accumulation of these substances, oxidative stress occurs, which leads to significant functional disorders since various components of cells may be damaged. The damage could include changes in protein structures, nitrogenous bases, and destruction of membranes (Powers and Jackson, 2008). Muscle cells contain endogenous cellular defense mechanisms in the form of enzymatic and non-enzymatic antioxidants that can partially eliminate ROS (Sen et al., 1994; Banerjee et al., 2003). A changes in the level of enzymes (*SOD GPx*, and *CAT*) under loading was observed in both animals and humans (Sen, 1995). During exhausted loads, the amount of *SOD* increased in skeletal muscle (Powers et al., 1999), whereas the level of *GPX* activity had not changed (Brady et al., 1979) or was increased (Powers et al., 1999). Similar phenomena were observed concerning *CAT* activity when no changes were observed during exercise (Powers et al., 1994a), and there was an increase in activity under certain loads (Ji and Fu, 1992). Thus, the understanding of the mechanisms involved in the process of increasing antioxidant enzymes during exhaustive exercise has yet to be under debate.

Intense muscle loading contributes to rise of oxidative stress in muscle tissue (Powers et al., 1999). The presence of ROS inhibits the tricarboxylic acids cycle and disrupts the mitochondria electronic transport chain (Janero and Hreniuk, 1996). With intense physical activity, the rate of hydrolysis of ATP may exceed the rate of its resynthesis, which leads to a decrease in ATP and the formation of muscle fatigue effects (Graham et al., 1978; Sahlin, 1985). However, the synthesis of ATP during training can be supported by antioxidants supplementation, which accelerate the process of muscle recovery after fatigue. There are evidences demonstrated a moderately beneficial effect of NAC (Supinski et al., 1997; Sandstrom et al., 2006) and β-Alanine (Stout et al., 2007; Ghiasvand et al., 2012; Summermatter and Handschin, 2012; Schnuck et al., 2016) supplementation for exercise with a substantial contribution from oxidative metabolism. It was shown unspecific antioxidant activity of NAC with increase GSH synthesis and reduce muscle-derived ROS levels during contraction (Sandstrom et al., 2006). Another study suggested a delay accumulation of lactate during exercise and increasing time to exhaustion under β-Alanine supplementation (Summermatter and Handschin, 2012). In our previous study, we showed a facilitation effect of water-soluble C_60_ fullerenes on the removal of some symptoms of skeletal muscle fatigue (Prylutskyy et al., 2017). So, we consider that because of novel field of C_60_ fullerenes application (muscle fatigue) it was important to assessed the adequacy and efficiency of C_60_ fullerene protective properties against oxidative damage in comparison with the action of known antioxidant NAC and β-Alanine. It was hypothesized that due to its unique chemical structure and bioactivity a water-soluble C_60_ fullerenes would have predominant influence on prooxidant-antioxidant homeostasis of rat muscle tissue. In our the previous work we detected the effect of the C_60_ fullerenes in the short-term period after intramuscular injection (Prylutskyy et al., 2017), in this study we determined the most optimal way of its application and investigated the effect of C_60_ fullerenes after preliminary administration, which implies its preventive use in case of muscular fatigue.

## Materials and Methods

### Material Preparation and Characterization

A highly stable C_60_FAS at a maximum concentration of 0.15 mg/ml was prepared as described earlier (Scharff et al., 2004; Ritter et al., 2015). Briefly, for the preparation of C_60_FAS we used a saturated solution of pristine C_60_ fullerene (purity >99.99%) in toluene with a C_60_ molecule concentration corresponding to maximum solubility near 2.9 mg/ml, and the same amount of distilled water in an open beaker. The two phases formed were treated in ultrasonic bath. The procedure was continued until the toluene had completely evaporated and the water phase became yellow colored. Filtration of the aqueous solution allowed to separate the product from undissolved C_60_ fullerenes.

### DLS Measurements

Measurements of the hydrodynamic size distribution for C_60_ fullerenes in aqueous solution were performed by dynamic light scattering (DLS) on a Zetasizer Nano-ZS90 (Malvern, Worcestershire, United Kingdom) at room temperature. A DLS instrument equipped with a HeNe laser (max 5 mW) operating at a wavelength of 633 nm was used. The measurements were performed at a 90° scattering angle. The autocorrelation function of the scattered light intensity was analyzed with Static Light Scattering software.

### Zeta Potential Measurements

Zeta potential measurements for C_60_FAS were carried out on a Zetasizer Nano-ZS90 (Malvern, Worcestershire, United Kingdom) at room temperature. The results were evaluated using the Smoluchowski approximation, which is known to be valid only for spherical-like particles.

### Procedure and Experimental Groups

Experiments were performed on male Wistar rats weighing 280–350 g with ages ranging from 4 to 6 months. The animals were purchased from a state-controlled animal farm through the common animal facility of Bogomoletz Institute of Physiology (Kyiv). The experimental animals were housed in Plexiglas cages (four rats per cage) and kept in an air-filtered and temperature-controlled (20–22°C) room. The rats received a standard pellet diet and water *ad libitum*. The present study was approved by the Ethics Committee of the Institute and performed according to the European Communities Council Directive of November 24, 1986 (86/609/EEC).

All animals were randomly divided into 7 groups: 1st – fatigue-induced animals (*n* = 6); 2nd – vehicle-injected [fatigue-induced rats with a preliminary intraperitoneal (i.p.) injection of 0.3 ml of saline solution, *n* = 6]; 3rd – C_60_FAS-injected [F-injection; fatigue-induced rats with a preliminary i.p. injection of 0.3 ml (0.14 mg/kg) of C_60_FAS, *n* = 6]; 4th – animals with oral administration of C_60_FAS [F-drinking; fatigue-induced rats with a preliminary (within 5 days, 0.225 mg/kg per day) oral introduction of C_60_FAS, *n* = 6]; 5th – NAC-injected (fatigue-induced rats with a preliminary i.p. injection of 150 mg/kg of NAC dissolved in saline solution, *n* = 6); 6th – β-Alanine-injected (β-Al-injection; fatigue-induced rats with a preliminary i.p. injection of 110 mg/kg of β-Alanine dissolved in saline solution, *n* = 6); and 7th – intact animals (rats were used only for biochemical studies, *n* = 6).

It should be noted that the toxicity of the pristine C_60_ fullerenes is strictly dependent on their size (the degree of aggregation in the water). In our previous works (Prylutska et al., 2009, 2017; Tolkachov et al., 2016), we investigated in detail the *in vitro* toxicity of C60FAS. We can conclude that at a maximum concentration of 0.15 mg/ml, C60FAS (the diameter of nanoparticles is up to 50 nm) does not exhibit any genotoxicity and cytotoxicity to various types of cells, including human ones. It is important to note that the doses of C_60_FAS used in this study do not present any acute or subacute toxicity in animals: they were significantly lower than the maximum tolerated dose of pristine C_60_ fullerene, which was found to be 5 g/kg both for oral or i.p. administration to rats (Gharbi et al., 2005). Toxic or lethal effects were not observed in the studies of the impact of C_60_ fullerenes after oral administration to rats at a total dose of 2 g/kg for 14 days (Mori et al., 2006). Moreover, according to our previous data (Tolkachov et al., 2016) C_60_FAS at the concentrations up to 24 μg/ml does not manifest any *in vitro* toxic effect on human mesenchymal stem cells. C_60_FAS (0.1 mg/ml) does not induce DNA strand breaks in the human lymphocytes as revealed by the comet assay (Prylutska et al., 2017). Finally, the authors (Świdwińska-Gajewska and Czerczak, 2016) state that oral exposure of pristine C_60_ fullerene nanoparticles does not lead to major adverse effects because they were not mutagenic and genotoxic in experimental research. The pristine C_60_ fullerene is characterized by low toxicity and it does not pose a risk in the occupational environment.

Fatigue of the *TS* of rat was induced by electrical stimulation of *n. tibialis*. C_60_FAS, NAC, β-Alanine or saline solution were administered 1 h prior to electrical stimulation. After the experiment, the *TS* of all animals in all groups were removed for biochemical analysis.

The animals were anesthetized with ketamine (100 mg/kg “Pfizer”, United States) combined with xylazine (10 mg/kg, “Interchemie,” Holland), tracheostomized and artificially ventilated (out of necessity). The left and right *TS* muscles were separated from the surrounding tissue, their tendons were detached at the distal insertions, and a small bone chip from the heel was left behind. The *n. tibialis* was separated from the tissue and cut proximally, and all branches of the nerve, except nerves innervating the *TS*, were cut. This nerve was mounted on a bipolar platinum wire electrode for electrical stimulation. The hindlimb muscles and nerves were covered with paraffin oil in a pool formed by the skin flaps. The ECG and heart rate were continuously monitored. Pools with mineral oil were maintained at 37–38°C using radiant heat. The *TS* muscle was connected via the Achilles tendon to the servo-control muscle puller. A linear motor under servo-control was used as the muscle puller. The muscle tension was measured by semi-conductor strain gauge resistors glued on a stiff steel beam mounted on the moving part of the linear motor. The stiffness of the puller exceeded 0.06 N/mm, whereas the time constants of the length transients did not exceed 60 ms.

**FIGURE 1:**
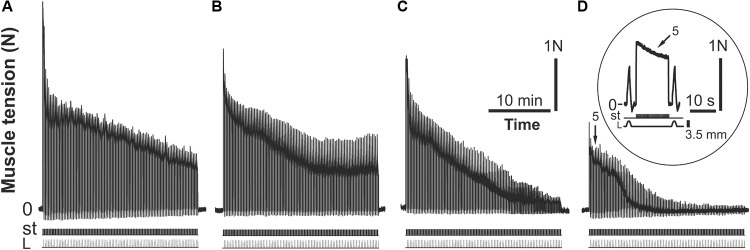
Strength of the *m. triceps surae* (*TS*) contractions during consecutive (I–IV) series of 30-min intermittent stimulations of the *n. tibialis*; the rat belongs to the fatigue-induced group of animals (group 1) **(A–D)**. The 5th individual tetanic contraction of *TS* in the IV stimulation series is presented in a circle at a higher time scale on **(D)**. P – muscle force (Newton, N), st – stimulation mark, L – muscle length (stretching, mm).

To induce muscle fatigue, a series (30 min duration) of intermittent high-frequency electrical stimulations was used (**Figures 1A–D**), separated by rest intervals of 15 min. Four stimulation series were used for the rats in group 1, and 5 stimulation series were used for the other groups of animals. Each series consisted of trains of 0.2 ms rectangular pulses at a rate of 40 s^-1^ at a 12.4 s duration, and the series were separated by 5 s intervals of rest (**Figure 1D**, in a circle). The stimulus current was set to 1.3–1.4 times higher than the motor threshold. At the end of the 12.4 s stimulation, the muscle was stretched, and the changes in length had a bell-shaped form (one period of 4 Hz sinusoidal signal with corresponding phase locking) with a 3.5 mm amplitude and 2 s duration (**Figure 1D**, bottom row in a circle). The muscle reaction to the stretching appeared to be a tension increase after continuous stimulation. These stretches were applied before the post-stimulation twitches to remove, or at least diminish, the after-effects remaining from continuous stimulation (Kostyukov et al., 2000). The command signal to the muscle puller was derived from a DAC and was adjusted by a scaling amplifier and low-pass filter (0–100 Hz). In parallel, two analog signals (muscle tension and length) and pulse signals (stimulation pulses) were sampled via corresponding ADC channels. The signals were collected by PC using an input-output interface device (CED Power 1401) with 12-bit resolution.

Data acquisition was performed using the program “Spike2” (CED). Input signals were digitized at rates of 5 kHz (muscle tension) and 1 kHz (other signals). Data analysis, including statistical treatment and graph plotting, was performed using the program Origin 8.0 (OriginLab Corp., United States).

### Biochemical Experiment

After acute exercise, the excised muscles (soleus and gastrocnemius) were rapidly dissected, free of fat and tendons were removed, and the muscles were divided into several portions and stored in liquid N_2_. For *GSH* (reduced glutathione) analysis, the tissue samples were transferred to a medium containing 1N perchloric acid (1:10 w/v) and homogenized with a motor-driven Potter-Elvehjem glass homogenizer. Resultant homogenate was centrifuged at 10,000 *g* for 10 min (4°C). *GSH* content was measured spectrophotometrically (Sedlak and Lindsay, 1968). For the activities of enzymes, *H*_2_*O*_2_ and lipid peroxidation assays, the muscle samples were thawed and homogenized in 50 mM phosphate buffer with 2 mM *EDTA* (pH 7.4) at 4°C (1:9 w/v). The homogenates were then centrifuged then for 15 min at 15,000 *g* (4°C), and the post-mitochondrial supernatant was stored at -70°C.

Oxidative damage in tissue was measured using a *TBARS* assay. *TBARS* were isolated by boiling tissue homogenates for 15 min at 100°C with thiobarbituric acid reagent (0.5% 2-thiobarbituric acid/10% trichloroacetic acid/0.63 M/dm^3^ hydrochloric acid) and measuring the absorbance at 532 nm. The results were expressed as nM *TBARS*/mg protein using 𝜀 = 1.56 × 10^5^ dm^3^/M/cm (Buege and Aust, 1978). The data on ROS formation were obtained from dichlorofluorescein (DCF) fluorescence. The tissue homogenates were loaded for 20 min at 37°C with non-fluorescent probe (2′,7′-dichlorodihydrofluorescein diacetate, DCFHDA) which is known to be decomposed in cells to give dichlorofluorescein upon oxidation by ROS, primarily hydroperoxide and superoxide anion. The final concentration of DCFH-DA was 10 μM. DCF formation was followed at the excitation wavelength of 488 nm and emission wavelength of 525 nm for 30 min by using a Hitachi F-2000 fluorescence spectrometer. The rate of DCFH-DA conversion to DCF was linear for at least 60 min, corrected with the autoxidation rate of DCFH-DA without protein. All assays were carried out in duplicates. Fluorescence was expressed as arbitrary fluorescence units.

*H*_2_*O*_2_ concentration in the tissue homogenates was measured using the FOX method, which is based on the peroxide-mediated oxidation of Fe^2+^, followed by a reaction of Fe^3+^ with xylenol orange (o-cresolsulfonephthalein 3′,3^′′^bis[methylimino] diacetic acid, sodium salt). This method is extremely sensitive and used to measure low levels of water-soluble hydroperoxide present in the aqueous phase. To determine the *H*_2_*O*_2_ concentration, 500 μL of the incubation medium was added to 500 μL of assay reagent (500 μm ammonium ferrous sulfate, 50 mm *H_2_SO_4_*, 200 μm xylenol orange, and 200 mm sorbitol). The absorbance of the *Fe^3^*^+^-xylenol orange complex (*A*_560_) was detected after 45 min. Standard curves of *H*_2_*O*_2_ were obtained for each independent experiment by adding variable amounts of *H*_2_*O*_2_ to 500 μL of basal medium mixed with 500 μL of assay reagent. The data were normalized and expressed as μm *H*_2_*O*_2_ per mg protein (Wolff, 1994).

Catalase activity was measured by decomposition of H_2_O_2_, which was determined by a decrease in absorbance at 240 nm (Aebi, 1983).

Reduced glutathione was determined using Ellman’s reagent. One milliliter of supernatant was treated with 0.5 ml of Ellman’s reagent (5.5′-dithio-bis-nitrobenzoic acid in abs. ethanol) and of 0.4 M *Tris HCl* buffer with 2 mM *EDTA*, pH 8.9. The absorbance was read at 412 nm in a spectrophotometer (Sedlak and Lindsay, 1968).

Manganese-superoxide dismutase (*Mn-SOD*) activity was estimated by the method of Misra and Fridovich (1972), which is based on the inhibition of autoxidation of adrenaline to adrenochrome by the *SOD* contained in the examined samples. The mitochondrial samples were preincubated at 0°C for 60 min with 6 mM *KCN*, which produces total inhibition of *Cu*, *Zn-SOD* activity. The results were expressed as the specific activity of the enzyme in units per mg protein. One unit of *SOD* activity was defined as the amount of protein causing 50% inhibition of the conversion rate of adrenaline to adrenochrome under specified conditions.

The activity of selenium-dependent *GPx* was determined according to the method of Flohé and Günzler (1984). Briefly, the reaction mixtures consisted of 50 mM *KPO_4_* (pH 7.0), 1 mM *EDTA*, 1 mM *NaN_3_*, 0.2 mM *NADPH*, 1 mM *GSH*, 0.25 mM *H*_2_*O*_2_, and 226 U/ml glutathione reductase, and the rates of *NADPH* oxidation followed at 340 nm.

Lactic acid was determined in muscle tissue after deproteinization with 6% (wt/wt) perchloric acid by sitting the tissue on ice for 15 min and then centrifuging the tissue at 14,000 *g* for 5 min. The supernatant was neutralized with 5 M *K_2_CO_3_*, clarified again to remove potassium perchlorate, and stored at -70°C. The assay mixtures contained glycine/*EDTA*/hydrazine hydrate buffer (pH 9.5), 0.05 mM *NAD*, 10 units of lactate dehydrogenase, and sample (100 μl), and the mixtures were incubated at 37°C for 20 min. The *LA* concentration was determined spectrophotometrically at 340 nm in a 1.0-ml total reaction volume (Hohorst, 1970).

Protein concentration was estimated with the Bradford method using bovine serum albumin as a standard. All chemicals were purchased from Sigma, Fluka and Merck and had the highest available purity.

### Statistical Analysis

In the electrophysiological study, each stimulation series was averaged (100 stimulations in one series). The average value of the first series was set to 100%, and the other series were normalized in relation to this and presented graphically for one of hindlimb. During experiments, it was found that till the moment of a drastic decrease of TS contraction force control animals maintain a certain force level of muscular contraction only during three series of electrical stimulation. To confirm the muscle fatigue factor, data from four stimulation series were taken for the study. Some animals from groups 3–6 to maintain a certain force level of muscular contraction until 5–6 series of electrical stimulation. Therefore, for further analysis, data from the first 5 series were taken.

Mean values (mean ± SD) of the *TS* muscle strength after C_60_FAS, NAC, β-Alanine, saline solution induction or without any induction were compared using two-way statistical analysis of variance (ANOVA). The factors of variation included two conditions: time and the effects of the C_60_FAS (NAC, β-Alanine or non-injected). A Bonferroni *post hoc* analysis was used to determine the differences between groups. The level of significance was set at *P* < 0.05.

Biochemical data are expressed as the means ± SEM for each group. The differences among experimental groups were detected by one-way analysis of variance (ANOVA) followed by Bonferroni’s multiple comparison test. Values of *P* < 0.05 were considered significant.

## Results

It has been established that the size of water-soluble C_60_ fullerene particles directly correlates with their cytotoxicity and biological properties (Lyon et al., 2006; Prylutska et al., 2009; Song et al., 2011; Lalwani and Sitharaman, 2013; Zhang et al., 2015). Depending on the size, water-soluble C_60_ fullerene particles can penetrate through the plasma membrane into the cell or be adsorbed on the surface of the membrane (Foley et al., 2002; Schuetze et al., 2011; Franskevych et al., 2017). In this regard, the main advantage of using pristine C_60_ fullerenes as powerful antioxidants (Gharbi et al., 2005; Prylutska et al., 2008) is their ability to be localized preferentially to mitochondria, which generates a substantial amount of cellular ROS (Foley et al., 2002; Youle and Karbowski, 2005). Thus, since the size of C_60_ fullerene particles and stability of their aqueous solution (the degree of aggregation in water) may influence their bioactivity, the DLS and zeta potential studies of C_60_FAS were performed.

### DLS and Zeta Potential Studies

The DLS results for investigated C_60_FAS clearly demonstrate that there was a monomodal nanoparticle size distribution in a (15–30) nm range, i.e., these nanoparticles in particular have specific bioactivity. This result is similar to our previous probe microscopic data, which directly correlate with C_60_ fullerene bioactivities (Prylutska et al., 2014; Skamrova et al., 2014).

The magnitude of the zeta potential is related to the stability of colloid dispersions because it determines the degree and nature of the interaction between the particles of the dispersal system. The value of zeta potential for C_60_FAS was equal to -23 mV, which agrees well with our previously published data (Ritter et al., 2015). A high negative charge for colloid nanoclusters (or, more strictly, the electrostatic repulsion between the negatively charged nanoclusters) seems to play a significant role in the stabilization of C_60_FAS (i.e., it disfavors the aggregation and makes the solution electrically stable).

### Electrophysiological Experiments

**FIGURE 2:**
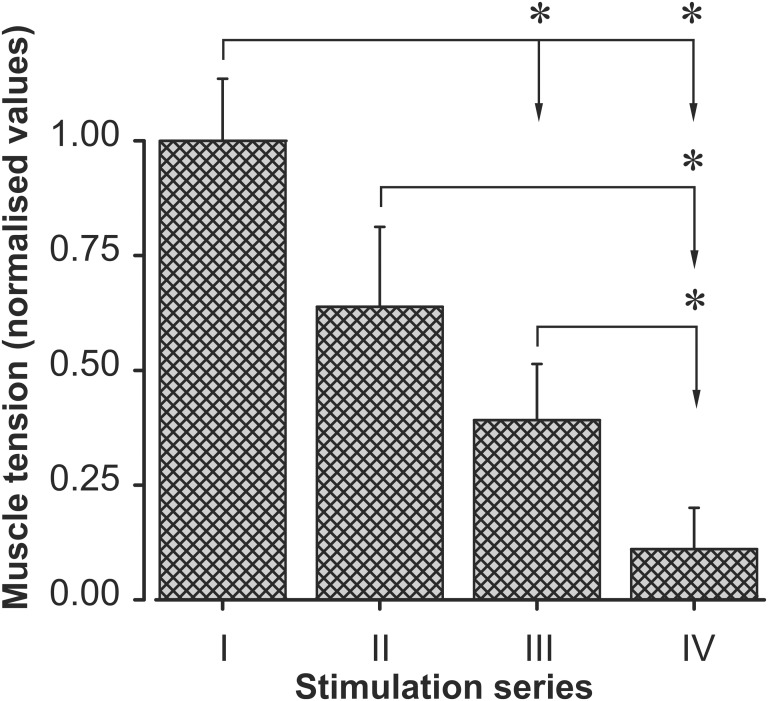
Changes in the *TS* muscle strength in the I–IV series of fatigue stimulation. There are normalized averaged force (mean ± SD) (normalization with respect to the first series) values. The averaging procedure has been applied to the data registered in six animals of the fatigue-induced group of rats (group 1), and the data were averaged with respect to the corresponding stimulation series. Asterisks denote significant differences (*P* < 0.05) between the muscle strength of different electrical stimulation series.

As a result of intermittent high-frequency electrical stimulation (30 min duration, 40 Hz) of *TS* muscle in the rats of the first and second groups during four series, the reduction of the force contraction was recorded until the muscle ceased to demonstrate clear contractions. **Figure 1** shows an example of the *TS* force response changes in one animal of the first group during fatigue development. In this case, the dynamics of changes in muscle force reflected fatigue development and a drop in amplitude in single contractions. In the first series of stimulations, a gradual reduction of *TS* activity level was observed over 30 min. In the second series of stimulations, which were started after a 15 min rest, the amplitudes of tetanic contractions were somewhat recovered; however, they did not reach the initial level, continuing to decrease. Within the third series of stimulations (after 90 min of electrical stimulation), an abrupt drop in the muscle force was observed. Within this time interval, the maximum significant strength level decrease (*P* < 0.05) was observed with respect to the first series of stimulations (**Figure 2**). After another interruption between stimulations, the isometric muscle force contraction continued to decline without further recovery. At the same time, such a decrease in muscle force response was statistically significant (*P* < 0.05) within the IV series of stimulations with respect to that one in the I, II, and III series (**Figure 2**). Note that statistically significant differences (*P* > 0.05) in the strength of muscle contractions between rats in groups 1 and 2 were not found.

**FIGURE 3:**
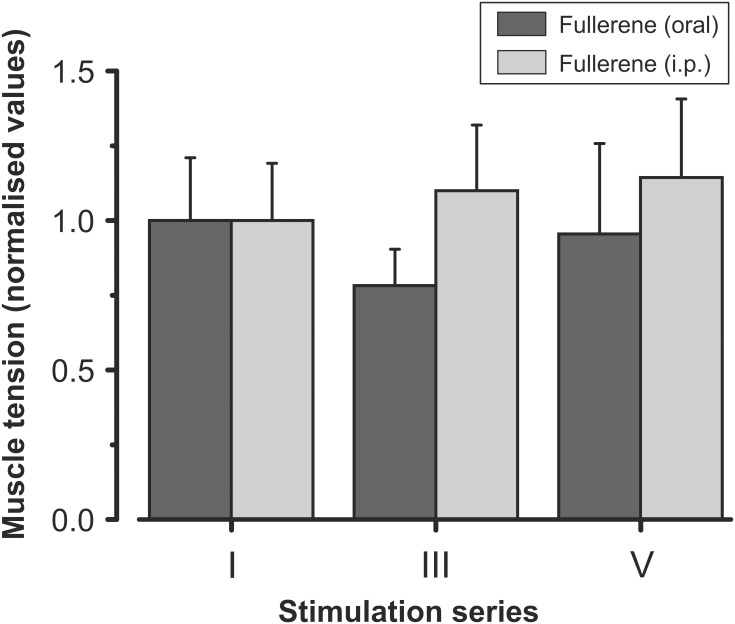
Averaged characteristics (mean ± SD) of the normalized (with respect to the first stimulation series) values of the muscle strength after preliminary intraperitoneal injection and oral administration of C_60_FAS (*dark gray* and *light gray bars*, respectively). I, III, and V – series of electrical fatiguing stimulation. Values of six animals in each group were averaged according to stimulation series. Note the absence of significant differences between data obtained in rats of groups 3 and 4.

For assessments of the effectiveness of administration method of the C_60_FAS under conditions of fatigue stimulation it was made a comparison of the changes in the force levels developed by muscles in animals with C_60_ fullerene i.p. injection (F-injection) and in animals that drank C_60_ fullerene for 5 days (F-drinking) (**Figure 3**). The analysis did not reveal any significant differences in the muscle strength after studying the effects of these substance administration methods on muscle fatigue (F-injection vs. F-drinking). In this case, under conditions of both F-injection and F-drinking, the muscle maintained the same constant force level during the first series of fatigue stimulation (30 min). In animals with a C_60_ fullerene i.p. injection during the third series of stimulations, the force level was slightly higher compared to the group of animals that drank C_60_FAS. Under conditions of further fatigue stimulation (V series) with F-injection and F-drinking, the difference in the level of the developed effort decreased (**Figure 3**). Thus, the absence of statistically significant differences (*P* > 0.05) made it possible for us to conduct further analyses of fatigue development using only F-drinking administration, since it is non-invasive and potentially more practical for future application.

**FIGURE 4:**
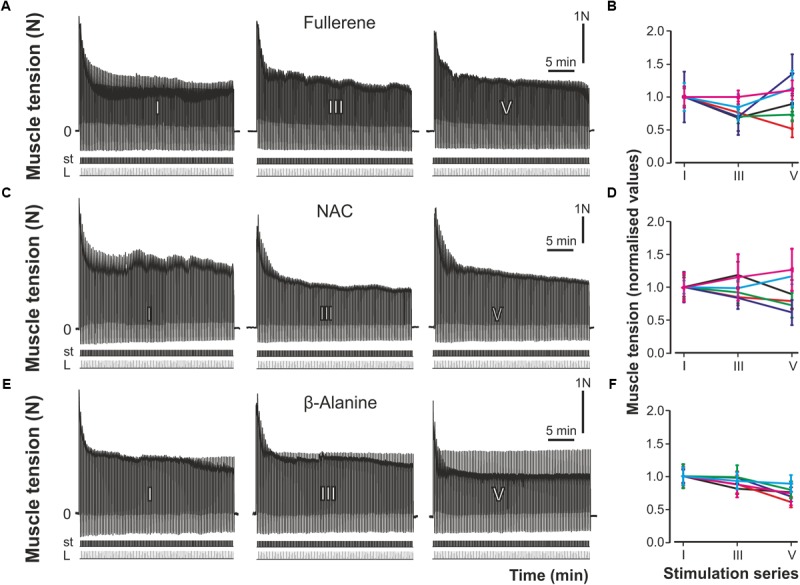
Strength of the muscle contractions after preliminary oral administration of C_60_FAS and injection of NAC and β-Alanine. **(A,C,E)** Protocol of registration of the muscle contractions after preliminary C_60_FAS oral administration, NAC- and β-Al-injection, respectively; **(B,D,F)** – results of six fatigue tests in the groups of animals with preliminary C_60_FAS oral administration, NAC- and β-Al-injection, respectively. I, III, and V – series of electrical fatigue stimulations, P – muscle force (Newton, N), st – stimulation mark, L – muscle length (stretching).

Further analysis suggested the force fatigue contractions on the background of a separate action of C_60_FAS attach be compared to exogenous antioxidants NAC and β-Alanine (**Figure 4**). The data obtained in these experiments (**Figures 4B,F**) indicate that a reduction in the developed force in the animals of the F-drinking, the NAC-injected and the β-Al-injected group was slower compared to fatigue-induced or vehicle-injected animals. Significant differences (*P* < 0.05) in muscle strength changes between control animals (groups 1 and 2) and experimental rats (groups 4–6) appeared after the second stimulation series. In groups 4–6, the muscle maintained a constant level of developed effort through the whole fatigue stimulation, which was demonstrated using native records of its force characteristics (**Figures 4A,C,E**). The animals of the F-drinking group held a stationary level of force longer than the NAC-injected and β-Al-injected animals. The dynamics of the force changes was similar in almost all animals of this group (**Figure 4B**). In the NAC-injected animals during the III series, there was decrease in the muscle strength amplitude along with further stabilization, but these effects were not significant (**Figure 4C**). It should be noted that in two animals of this experimental group, recovery of the muscle contraction force level to the force level at the initial fatigue stimulation stages occurred (**Figures 4C,D**). At the same time, in the F-drink group during the V series of fatigue stimulation, muscle strength recovery was also observed, and in some animals, its increase was recorded (**Figure 4B**). In the β-Al-injected group, the developed force was maintained at a steady-state level within each series of stimulations (**Figure 4E**). At the end of the I series of stimulation, there was a slight decrease in muscle force with some recovery in the III series of stimulations. However, during further stimulation (V series), the level of the muscle force was slightly lower compared to previous series (**Figure 4E**). In this case, in all animals of this experimental group a similar change in muscle force was observed (**Figure 4F**).

**FIGURE 5:**
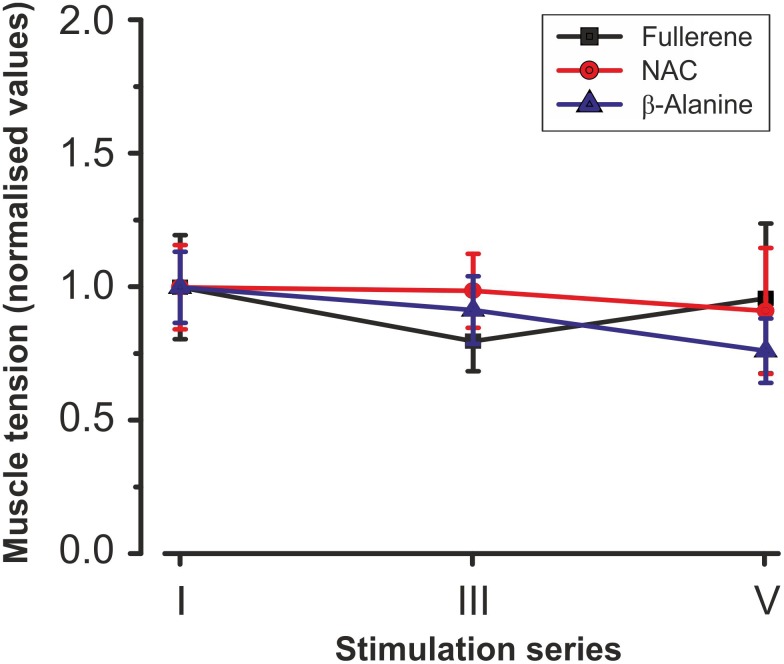
Averaged characteristics (mean ± SD) of normalized (with respect to the average values in the first stimulation series) values of the muscle strength after preliminary C_60_FAS oral administration, NAC- and β-Al-injection. Values of six animals in each group were averaged by stimulation series. Note the absence of significant differences between the data obtained in the rats of these groups. I, III, and V – a series of electrical fatigue stimulations.

To compare the action of the C_60_FAS and antioxidants on the force of contractions during fatigue development, a statistical analysis was performed (**Figure 5**). In NAC-injected animals, during the initial series of stimulation, there was maintenance of the force at a constant level followed by some decrease. The characteristics of fatiguing muscle contractions were somewhat different in the F-drinking group. In these experiments, the level of muscle force, after some initial reduction in the I series of stimulations, was further recovered and almost reached the control values. In the β-Al-injected group, a gradual decrease of the muscle force was observed. However, it should be noted that there was no significant difference in the muscle force for series conducted within the same animal group, as well as between groups (NAC-injected, F-drink, β-Al-injected) (**Figure 5**).

### Biochemical Experiments

**FIGURE 6:**
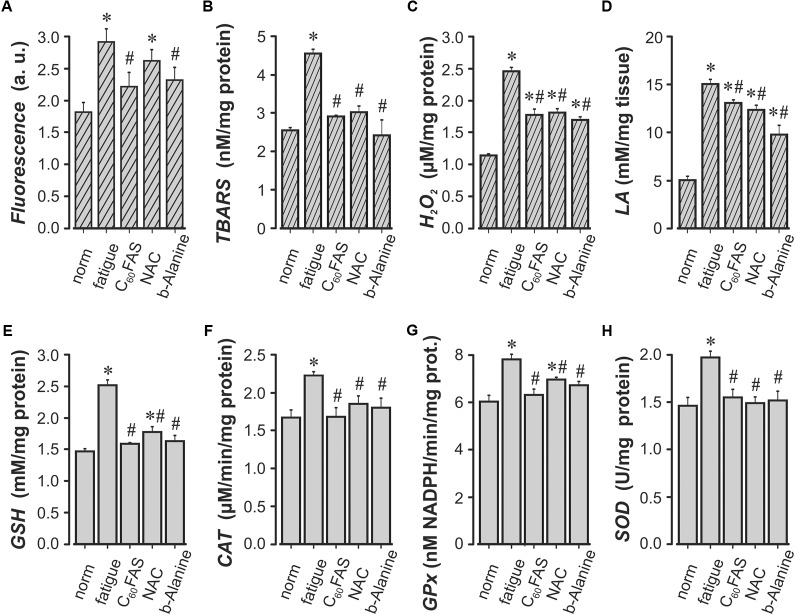
Indicators of the prooxidant-antioxidant balance in the *m. triceps surae* (*TS*) of rats. ROS formation **(A)**, the concentration of thiobarbituric acid reactive substances (*TBARS*) **(B)**, hydrogen peroxide (*H*_2_*O*_2_) **(C)**, lactic acid (*LA*) **(D)**, reduced glutathione (*GSH*) **(E)**, catalase (*CAT*) **(F)**, glutathione peroxidase (*GPx*) **(G)** and superoxide dismutase (*SOD*) **(H)** are in intact animal muscles (norm), *TS* fatigue-induced animals (fatigue) and *TS* fatigue-induced animals after C_60_FAS oral administration (C_60_FAS), NAC-injection (NAC) and β-Al-injection (β-Alanine), respectively. Values are the means ± SEM, *n* = 6. ^∗^*P* < 0.05 vs. “norm”; ^#^*P* < 0.05 vs. “fatigue.”

After the fatiguing tests, accumulation of secondary products and changes in antioxidant levels in the muscle tissues were determined (**Figure 6**). The obtained data clearly demonstrate the increased level of metabolic product (*LA*), markers of peroxidative and oxidative stress (*TBARS*, *H*_2_*O*_2_) and ROS formation after stimulation that indicates the occurrence of muscle fatigue (**Figure 6**). This increase was statistically significant with respect to the intact muscles (“*norm”*) and consisted of 61% for ROS formation, 78% for *TBARS*, 115% for *H*_2_*O*_2_, and 198% (*P* < 0.05) for *LA*. In turn, in response to these changes in the working muscle, there was a significant (*P* < 0.05) increase in endogenous antioxidant activity, including *GSH* (71%), *CAT* (18%), *GPx* (28.5%), and *SOD* (34%).

In animals that drank C_60_FAS, ROS formation and concentrations of *TBARS*, *H*_2_*O*_2_ and *LA* were significantly lower at 24.1, 36.1, 27.8, and 13.1% (*P* < 0.05), respectively, compared to the “*fatigue”* group. Similar changes in the level of these marker concentrations were also observed in the NAC-injection and β-Al-injection groups. A similar dynamic has been revealed in changes in the activity of antioxidant enzymes. Under conditions of fatigue development, the level of *GSH*, *CAT*, *GPx*, and *SOD* activity significantly increased by 71.4, 18.6, 28.5, and 34.2% relatively to the “*norm,”* respectively. The animal groups F-drinking, NAC-injection and β-Al-injection showed a decrease in endogenous antioxidants activity relative to the “*fatigue”* group (*P* < 0.05). Furthermore, in rats that drank C_60_FAS, the activity levels of *GSH*, *CAT*, and *GPx* were smaller.

## Discussion

In this study, we compared the effects of an aqueous colloid solution of pristine C_60_ fullerene with the action of the NAC and β-Alanine on exercise tolerance and contractile properties of rat *TS* during development muscle fatigue. This experimental approach allowed us to analyze and compare the characteristics of the force parameter changes of muscle contraction under conditions with the same fatigue stimulation pattern applied after oral introduction of C_60_FAS or separate i.p. injections of C_60_FAS, NAC and β-Alanine. The results showed a statistically significant reduction in muscle force in all animals without prior administration of C_60_FAS, NAC, and β-Alanine (**Figure 2**). To maintain the normal functional and physiological state of the muscle during the dynamic work performance, the duration of its recovery and active rest are very important factors (Harris and Sale, 2012). An insignificant decrease in the muscle contraction force compared to the control occurred in animals that drank C_60_FAS and in both NAC- or β-Al-injected rats (**Figure 5**). This outcome supported our previous finding that fullerene can lower the effects of fatigue development and promote force maintenance at a constant level (Prylutskyy et al., 2017). At the same time, in most animals of the F-drinking group, there was a recovery of the force level after some decrease, whereas in the NAC- and β-Al-injected groups, only an insignificant decline occurred (**Figures 4B,D,F**). A definite recovery of the contractile ability was noticed in only two animals from the NAC-injected group (**Figure 4D**). These data on the effects of C_60_FAS as a potent antioxidant under fatigue development conditions are in accordance with the data that were obtained earlier (Prylutskyy et al., 2017). In this case, one can speak about the predominant influence of C_60_ fullerene on muscle strength characteristics during fatigue development compared to the action of other investigated exogenous antioxidants, such as NAC or β-Alanine.

In our study, we showed that fatigue stimulation in the working muscle led to an increase in the metabolic products (*LA*) and the intensification of the oxidative processes, a namely a significant increase in ROS formation and lipid peroxidation, which occurs simultaneously with an increase in *CAT* and *GSH* activity in both fast and slow twitch muscles fibers relative to the intact muscle (**Figure 6**). The increased *LA* level further reduced the pH, which could induce various biochemical and physiological effects during muscular contractions, including glycolysis, phosphofructokinase, and calcium release (Wang et al., 2012). Therefore, *LA* is an important marker for evaluate the degree of fatigue of a living organism. In the group of animals that drank C_60_FAS, attenuation of oxidative stress was observed (i.e., a decrease in ROS generation and TBARS concentration). This outcome was confirmed by the data obtained earlier on the effects of C_60_ fullerenes for the prooxidant–antioxidant homeostasis of rat muscle tissue (Prylutskyy et al., 2017). Similar changes in the number of metabolites and peroxide oxidation markers were observed in the NAC-injected and β-Alanine-injected groups. It has been supported that β-Alanine supplementation increases carnosine levels and decreases lactate responses after high-intensity exercise in rat muscles (Culbertson et al., 2010). In skeletal muscles, carnosine acts as a pH buffer and functions as an antioxidant (Boldyrev et al., 1993), which suggests that there is a potential role for carnosine for reversing or limiting the effects of oxidative stress and cellular senescence. Therefore, it cannot be excluded that the β-Alanine intake results in decrease of lactate and H^+^-ion production. The skeletal muscles from NAC-injected rats showed a low content of *TBARS*, suggesting a reduced muscle fiber disruption due to cell membrane lipid peroxidation as compared with non-injected rats. This protective effect of NAC is due to direct scavenging of ROS and *GSH* synthesis enhancement (Aruoma et al., 1989; Supinski et al., 1997; Sen and Packer, 2000).

The important role of *GSH* in protecting against exercise-induced oxidative stress has been demonstrated in several studies (Sen et al., 1994; Leeuwenburgh et al., 1997). The increased amount of *GSH* in the stimulated muscle (without the C_60_FAS, NAC, or β-Alanine administration and after their application) indicates compensatory activation of the endogenous *GSH* antioxidant system on the action of the stimulus (**Figure 6E**). In some studies, it has been shown that during intense loads, there is a significant decrease in *GSH* content in *m. soleus* and an increase in *GSH* content in *m*. *deep vastus lateralis* (*DVL*), but did not alter *GSH* status in the liver or plasma (Leeuwenburgh et al., 1997). Although other studies indicate an increase in the concentrations of *GSH* and *GSSG* in *m. soleus*, less in *DVL* and *m. superficial vastus lateralis* (*SVL*). However, the *GSSG*/*GSH* ratio does not change significantly because *GSSG* can be reduced to *GSH* by glutathione reductase. Furthermore, exercising skeletal muscles appear to increase GSH import from plasma (Ji et al., 1992) as well as the synthesis of *GSH* in the liver from endo- or exogenous amino acids, which adds most of the circulating *GSH* (Meister and Anderson, 1983), that ensures plasma GSH homeostasis despite enhanced tissue *GSH* use. Our experiments showed an increase in *GSH* activity during fatigue and a decrease in *GSH* under the actions of C_60_FAS, NAC, or β-Alanine. This outcome is consistent with the data of other authors who have shown that the use of NAC, in particular, decreases exercise-induced *GSSG*, and blood lipid peroxidation in rats (Sen et al., 1994) improves muscle contractile functions as well as reduces low frequency fatigue in the diaphragm muscle (Shindoh et al., 1990) and human leg muscles (Reid et al., 1994).

An increase in the level of *H*_2_*O*_2_ activity during muscle contractions led to an increase in the *CAT* enzyme concentration (**Figure 6F**). These data also support studies performed earlier in rats (Ji and Fu, 1992; Hollander et al., 1999). It was shown that CAT activities were significantly elevated after exhaustive exercise with or without hydroperoxide injection in muscle and not in liver (Ji and Fu, 1992). Previously, it was reported that after the acute stage of the exercise, *CAT* was significantly higher in *m. soleus* than in *DVL* and *SVL*. Furthermore, the exercise at moderate intensities elicited significant increases in *CAT* activity in *DVL* (Ji et al., 1992). However, some studies have reported no changes in muscle *CAT* with training (Powers et al., 1994a; Radák et al., 1995), and a few studies even reported a decrease in *CAT* activities in *m. soleus* of the adult and old rats (Leeuwenburgh et al., 1994). In our study, during fatigue under NAC or β-Alanine treatment, the effects of *CAT* activity decreased with respect to “*fatigue,”* but exceeded the control values. However, in animals that drank C_60_FAS, *CAT* activity remained at the control level, which indicated that there was a greater compensatory effect of C_60_ fullerenes.

An increase in the concentration of oxidative process markers led to an increase in the activity of *GPX* in the working muscle (**Figure 6G**). However, with using of NAC and β-Alanine, the activity of this enzyme decreased relatively to “*fatigue,”* and in the group of F-drinking, it was practically restored to normal. Data about *GPX* activity remain controversial. Powers et al. (1994a) showed an increase in *GPX* activity in red *m. gastrocnemius* after endurance training in rats, whereas *m. soleus* and white *m. gastrocnemius* revealed no training effect. The magnitude of the GPX increase was directly related to exercise duration but independent of intensity. Thus, the *GPX* activity has demonstrated variable responses to acute exercise for various types of skeletal muscles. For example, *GPX* activity increased the next day after running on the treadmill until exhaustion in the *m. soleus* of rats, but not in *m. tibialis* (Radák et al., 1995). In our study, the activity level of *GPX* under fatigue conditions in the F-drinking group may indicate that C_60_FAS, by affecting endogenous antioxidants, prevents fatigue in an actively contracting muscle better than NAC and β-Alanine.

It has been proved that C_60_ fullerenes normalize cellular metabolism and nervous processes by increasing resistance to stress, increase the activity of enzymes and regenerative capacity of tissues, and also exhibit pronounced anti-inflammatory and antiallergenic effects (Cataldo and Da Ros, 2008) and effectively regulate the ATPase activity of actomyosin (Andreichenko et al., 2013). It has been experimentally found that C_60_ fullerenes and their derivatives may be auxiliary agents in complex therapy due to their ability to intensify the protective functions of the immune and antioxidant systems of the body (Ashcroft et al., 2006; Didenko et al., 2013; Halenova et al., 2016). In this regard, the above electrophysiological and biochemical results suggest a real perspective for the use of water-soluble pristine C_60_ fullerenes as potential agents for improving the efficiency of human skeletal muscle functioning by modifying ROS-dependent mechanisms that play an important role in the development of muscle fatigue.

It was shown that under intense loads in skeletal muscles, the activity of *SOD* increases (Ji and Fu, 1992; Ji et al., 1992; Lawler et al., 1993). We investigated the isoenzyme located in the mitochondria and *Mn^2^*^-^ contained in the active center (Weisiger and Fridovich, 1973). Under the conditions of the fatiguing tests, the activity of *Mn-SOD* increased, and after application of the test substances, it decreased approximately to same level, insignificantly exceeding the control value. These results were confirmed by the data of Radák et al. (1995), where it was shown that in rats after the exhausted treadmill run, the immunoreactive content and activity of both isoenzymes of *SOD* (*Mn-SOD* and *Cu*, *Zn-SOD* are found in the cytoplasm, in erythrocytes and liver (McCord and Fridovich, 1969) increased in the *m. soleus* and *m. tibialis* immediately after the run. In animals that had been previously injected by the antioxidant, attenuation of oxidative stress was observed (i.e., a decrease in *TBARS* concentration). An increase in *Mn-SOD* was noted even in 1 day after the load in hepatic tissue (Radák et al., 1996). Additionally, it was shown that during intense swimming training, myocardial and diaphragmatic *SOD* were induced in rats (Powers et al., 1993, 1994b). Ohishi et al. (1997) showed that an adequate physical endurance load increases both the activity and the content of *Mn-SOD* and that untrained rats are very sensitive to oxidative stress during exercise. *Mn-SOD* is a reliable indicator of physical condition. It is concluded that the muscles can react to the load in such way to reduce the damage that results from the accumulation of free oxygen radicals due to increased metabolic activity.

## Conclusion

It should be noted that there are studies in humans and animals demonstrating both various exogenous antioxidants and submaximal long-term training. The training increases the activity of endogenous antioxidants (Powers et al., 1993, 1994a) and the antioxidant defense system of glutathione (Banerjee et al., 2003) in skeletal muscles, which reduces the risk of cell damage, improving performance, and slowing down the muscle fatigue. Recent studies show that the addition of some antioxidant nutrients is necessary for physically active people (Sen, 1995; Powers et al., 1999; Banerjee et al., 2003).

The results obtained in this study confirm previous data on the mechanism of action of C_60_FAS intramuscular injections (Prylutskyy et al., 2017). A comparative analysis was produced using a non-invasive method for C_60_FAS administration, demonstrating a similarity of action of C_60_FAS with effects of already known exogenous antioxidants NAC and β-Alanine. But due to its membranotropic activity and the lack of acute *in vivo* toxicity (at least at low physiological concentrations), C_60_ fullerene has a greater effective influence on the homeostasis of muscle tissue in rats, which maintains the normal physiological state of the muscle and increases the duration of its active work.

## Author Contributions

IV, NB, and AM designed and performed the experiments, and the *in vitro* assays were performed by OG. UR and YP were responsible for C_60_FAS creation and characterization. WM and TT helped with preparation of the manuscript and provided funding support. DN and IM helped to collect and analyzed the data. YP and AK provided supervision and guidance throughout this study. All authors revised the manuscript for important intellectual content and approved the final version of the article.

## Conflict of Interest Statement

The authors declare that the research was conducted in the absence of any commercial or financial relationships that could be construed as a potential conflict of interest.

## Abbreviations

C_60_FAS: pristine C_60_ fullerene aqueous colloid solution
CAT: catalase
GPx: glutathione peroxidase
GSH: reduced glutathione
H_2_O_2_: hydrogen peroxide
NAC: antioxidants *N*-acetylcysteine
SOD: superoxide dismutase
TBARS: thiobarbituric acid reactive substances
TS: *m. triceps surae*
